# Adenosine Kinase Inhibition Prevents Severe Acute Pancreatitis *via* Suppressing Inflammation and Acinar Cell Necroptosis

**DOI:** 10.3389/fcell.2022.827714

**Published:** 2022-02-23

**Authors:** Shukun Sun, Yu Han, Chuanxin Zhang, Han Liu, Bailu Wang, Shengchuan Cao, Qiuhuan Yuan, Shujian Wei, Yuguo Chen

**Affiliations:** ^1^ Department of Emergency and Chest Pain Center, Qilu Hospital, Cheeloo College of Medicine, Shandong University, Jinan, China; ^2^ Clinical Research Center for Emergency and Critical Care Medicine of Shandong Province, Qilu Hospital, Cheeloo College of Medicine, Institute of Emergency and Critical Care Medicine of Shandong University, Shandong University, Jinan, China; ^3^ Key Laboratory of Emergency and Critical Care Medicine of Shandong Province, Key Laboratory of Cardiopulmonary-Cerebral Resuscitation Research of Shandong Province, Qilu Hospital, Cheeloo College of Medicine, Shandong University, Jinan, China; ^4^ The Key Laboratory of Cardiovascular Remodeling and Function Research, The State and Shandong Province Joint Key Laboratory of Translational Cardiovascular Medicine, Chinese Ministry of Education, Qilu Hospital, Cheeloo College of Medicine, Chinese Ministry of Health and Chinese Academy of Medical Sciences, Shandong University, Jinan, China; ^5^ Clinical Trial Center, Qilu Hospital, Cheeloo College of Medicine, Shandong University, Jinan, China

**Keywords:** adenosine kinase, inflammation, necroptosis, endoplasmic reticulum stress, adenosine A2A receptor

## Abstract

**Background:** Inflammatory disorder and acinar cell death contribute to the initiation and progression of severe acute pancreatitis (SAP). Adenosine kinase (ADK) has potential effects on both inflammation and cell death. However, the role of ADK in SAP remains to be explored.

**Methods:** To establish an experimental SAP model, male C57BL/6 mice were intraperitoneally injected with cerulein (50 μg/kg, seven doses at hourly intervals) and LPS (10 mg/kg, at the last cerulein injection). For ADK inhibition, ABT702 (1.5 mg/kg) was intraperitoneally injected 1 h before cerulein treatment. The pancreas and serum were collected and analyzed to determine the severity of pancreatic injury and explore the potential pathophysiological mechanisms. Pancreatic acinar cells (AR42J) were used to explore the *in vitro* effects of ADK inhibition on cerulein–induced inflammation and necroptotic cell death.

**Results:** ADK inhibition notably attenuated the severity of SAP, as indicated by the decreased serum amylase (7,416.76 ± 1,457.76 vs. 4,581.89 ± 1,175.04 U/L) and lipase (46.51 ± 11.50 vs. 32.94 ± 11.46 U/L) levels and fewer pancreatic histopathological alterations (histological scores: 6.433 ± 0.60 vs. 3.77 ± 0.70). MOMA-2 and CD11b staining confirmed that ADK inhibition prevented the infiltration of neutrophils and macrophages. The phosphorylation of nuclear factor-κB (NF-κB) was also reduced by ADK inhibition. ADK inhibition markedly limited the necrotic area of the pancreas and prevented the activation of the necroptotic signaling pathway. Endoplasmic reticulum (ER) stress was activated in the pancreas using the SAP model and cerulein–treated AR42J cells whereas ADK inhibition reversed the activation of ER stress both *in vivo* and *in vitro*. Moreover, the alleviating effects of ADK inhibition on ER stress, inflammation, and cell necroptosis were eliminated by the adenosine A_2A_ receptor antagonist.

**Conclusion:** ADK inhibition reduced inflammation and necroptotic acinar cell death in SAP *via* the adenosine A_2A_ receptor/ER stress pathway, suggesting that ADK might be a potential therapeutic target for SAP.

## Introduction

Acute pancreatitis (AP) is the leading cause of gastrointestinal disease-related hospital admission and causes considerable morbidity and mortality (Lee and Papachristou, 2019; Mayerle et al., 2019). Due to improvements in the timely and accurate diagnosis of AP and the care of severe patients, AP-related mortality has decreased from 1.6% to 0.8% over the past decade in the United States (Lee and Papachristou, 2019). However, up to 40% of worldwide patients with AP develop new-onset prediabetes or diabetes while 25% of patients develop exocrine pancreatic insufficiency (Das et al., 2014; Hollemans et al., 2018). Moreover, the global incidence of AP is increasing (Petrov and Yadav, 2019). Therefore, AP, especially severe acute pancreatitis (SAP), continues to cause substantial morbidity and sequelae and represents a great socioeconomic burden.

Inflammatory disorder and acinar cell death are the major hallmarks of AP (Bhatia, 2004b; Habtezion, 2015). During AP, pancreatic acinar cells produce tumor necrosis factor (TNFα), interleukin-6 (IL-6), monocyte chemoattractant protein-1 (MCP-1), and many other proinflammatory molecules (Habtezion, 2015). Rapid neutrophil infiltration and subsequent macrophage recruitment are also activated and play essential roles in the progression of AP (Shrivastava and Bhatia, 2010; Yang et al., 2015). In addition to inflammation, acinar cells undergo two major modes of death during AP: necrosis and apoptosis (Wang et al., 2016). Mild acute pancreatitis is associated with acinar cell apoptosis, while SAP involves extensive acinar cell necrosis and is inversely correlated with the rate of apoptosis (Bhatia, 2004b; Mayerle et al., 2019). Necrosis has long been considered unregulated and passive cell death; however, necroptosis is a new regulated form of necrosis, and its emerging role in SAP has attracted increasing attention (Wang et al., 2016).

Adenosine kinase (ADK) phosphorylates adenosine to adenosine monophosphate (AMP) and is the principal enzyme in determining the intracellular and extracellular adenosine levels (Boison, 2013). As an endogenous purine ribonucleoside, adenosine exerts widespread physiological and pathophysiological functions as a homeostatic and metabolic regulator in all living systems (Allard et al., 2020). ADK inhibition increases both the intracellular and extracellular adenosine levels. Increased adenosine reduces the activation of leukocytes and macrophages and suppresses the proinflammatory response (Hasko and Cronstein, 2013; Xu et al., 2017). Recently, we found that ADK inhibition prevents reperfusion-induced cardiomyocyte apoptosis and necroptosis (Wang et al., 2021). Moreover, ADK might also play a role in acinar cell necroptosis during SAP.

In the present study, we sought to determine whether ADK plays a role in SAP by regulating inflammation and cell necroptosis. Using mouse models, we investigated the effects of ADK on acute pancreatitis and found that ADK inhibition attenuates the severity of AP by limiting the infiltration of neutrophils and macrophages, suppressing the production of proinflammatory molecules and preventing acinar cell necroptosis.

## Materials and Methods

### Reagents and Antibodies

Cerulein was purchased from MedChem Express (HY-A0190, Shanghai). Lipopolysaccharide (LPS) was purchased from Sigma (L2630, Shanghai). ABT702 was purchased from Merck Millipore (116890, Shanghai). ZM241385 (an adenosine A_2A_ receptor antagonist) and GSK2606414 (PERK inhibitor) were purchased from Selleck (S8105 and S7307, respectively, Shanghai). The following primary antibodies were purchased: *ß*-actin antibody (Proteintech, 66009), ADK antibody (Abcam, ab227087), RIP1 antibody (CST, 3,493), phosphor-RIP1 (Ser161) antibody (Affinity, AF7377), RIP3 antibody (CST, 15828), phosphor-RIP3 antibody (Abcam, ab195117), mouse MLKL antibody (Proteintech, 66675), rat MLKL antibody (Abcam, ab243142), phosphor-MLKL antibody (Affinity, AF7420), NF-κB p65 antibody (CST, 8242), phosphor-NF-κB p65 antibody (CST, 3,033), GRP78 Antibody (Proteintech, 11587), CHOP antibody (Proteintech, 15204), PERK antibody (Proteintech, 24390), phosphor-PERK antibody (CST, 3,179), eIF2α antibody (CST, 5324), phosphor-eIF2α antibody (CST, 3,398), CD11b antibody (Abcam, ab133357), MOMA-2 antibody (Abcam, ab33451), and amylase antibody (Santa Cruz Biotechnology, sc-46657). Secondary antibodies were purchased from Cell Signaling Technology (Danvers, MA, United States).

### Mouse Acute Pancreatitis Model

Male wild C57BL/6 mice (8 weeks) were provided by the Experimental Animal Facility of Shandong University. The mice were fed a chow diet and provided water ad libitum. To establish an SAP model, the mice were fasted overnight and then intraperitoneally injected with 50 μg/kg cerulein (dissolved in DMSO, 20 μg/ml) every hour for a total of seven injections. After the seventh cerulein injection, the mice were immediately intraperitoneally injected with 10 mg/kg LPS (dissoved in saline water, 2.5 mg/ml). For ADK inhibition, 1.5 mg/kg ABT702 (dissolved in DMSO, 1 mg/ml) was intraperitoneally injected 1 h before the first cerulein injection. The mice were euthanized by neck dislocation 24 h after the first cerulein injection. Blood and pancreases were both collected for experimental analysis. This study was performed following the Guide for the Care and Use of Laboratory Animals and approved by the Institutional Animal Care and Use Committee of Qilu Hospital, Shandong University.

### Cell Culture

Pancreatic acinar AR42J cells were obtained from Procell (CL-0025, Wuhan, Hubei, China) and cultured in Ham’s F-12K medium supplemented with 20% fetal bovine serum and antibiotics (100 units/ml penicillin and 100 μg/ml streptomycin). Murine pancreatic acinar MPC-83 cells were purchased from Qcheng Bio (QC-0518, Shanghai, China) and cultured in RPMI-1640 medium supplemented with 10% fetal bovine serum. Both kinds of cells were grown in a humidified incubator with 95% air/5% CO_2_ at 37°C. For cerulein stimulation, AR42J or MPC-83 cells were incubated with cerulein (100 nM) for 24 h. For ADK inhibition, ABT702 (1 μM) was used 30 min before the first cerulein incubation. For adenosine receptor inhibition, ZM241385 (100 nM) was used 30 min before ABT702 incubation. For PERK inhibition, GSK2606414 (5 μM) was used 30 min before cerulein incubation.

### Histopathological Examination and Scoring

Samples of the pancreases collected from the mice were fixed with 4% paraformaldehyde for more than 24 h. The tissues were then paraffin-embedded and cut into 5 μm—thick sections. After dewaxing and rehydration, hematoxylin-eosin staining was performed. The morphological study was performed under a light microscope by a pathologist who was blinded to the experiments. The Schmidt score was used to measure the pancreatic histopathology changes (Schmidt et al., 1992).

### Necroptosis Assay

Cell necroptosis in the pancreas was detected using double staining with Evans blue dye (EBD) and amylase antibody (Santa Cruz Biotechnology, sc-46657). EBD (10 mg/ml) was injected intraperitoneally for 4 h before the mice were euthanized. After the operation, the pancreas was separated and frozen in liquid nitrogen, and then cryosections of the pancreas were immunostained with an amylase antibody. Sections were observed and imaged by a fluorescence microscope (Olympus) and analyzed using ImageJ software (National Institutes of Health, Bethesda, MD, United States).

### Serum Lipase and Amylase Analysis

The *a*-amylase assay kit and lipase assay kit were from Nanjing Jiancheng Bioengineering Institute. Blood was collected through the cardiac apex. Then the blood was centrifuged at 1,500 g and 4°C for 15 min, and serum was collected for determination of *a*-amylase and lipase using the respective assay kits.

### Immunochemistry and Immunofluorescence Staining

After routine dewaxing and hydration, sections were stained with a primary antibody directed against CD11b (Abcam, ab133357). Then, sections were incubated in biotinylated secondary antibodies, followed by incubation with DAB as the chromogenic substrate. The stained sections were counterstained with hematoxylin. Immunofluorescence staining of MOMA-2 (Abcam, ab35541) was also performed in paraffin sections. After deparaffinization, hydration, antigen retrieval, and serum blocking, the sections were incubated overnight at 4°C with rat anti-MOMA-2 (1:50). Sections were washed with TBST 3 × 10 min and incubated with the species-specific secondary antibody anti-rat IgG H&L Alexa Fluor 594 (Abbkine, A23440) at room temperature for 2 h. Nuclear staining was performed with 4′, 6-diamino-2- phenylindole (DAPI, Abcam, ab104139). Representative images were captured with an Olympus BX63 microscope under a light or fluoresce pattern and analyzed using ImageJ software.

### Western Blot Analysis

Proteins were extracted using a mixture of a protease inhibitor, phosphatase inhibitor, EDTA BOSTER, AR1182, Wuhan, China) and RIPA lysate (Beyotime, Shanghai, China) in a 3:1:1:100 ratio. Then, protein concentrations were determined by Bicinchoninic Acid Assay. Proteins were separated by SDS-PAGE and transferred to polyvinylidene fluoride (PVDF) membranes (Merck Millipore, Billerica, MA, United States), which were blocked with Quick Block™ Blocking Buffer for 15 min at room temperature and incubated with primary antibodies at 4°C overnight. The membranes were incubated with horseradish peroxidase (HRP)-coupled secondary antibodies after washing with TBST 3 times. Then the membranes were visualized by chemiluminescence reagents and detected with Amersham Imager 600 (GE, Boston, United States). ImageJ software was used to analyze the intensity of the bands.

### Statistical Analysis

All data were analyzed using GraphPad Prism version 6.0. The data are presented as the mean ± SEM. Data have passed normality or equal variance tests. Student’s t-test or one-way analysis of variance followed by Tukey’s post hoc test were used to compare statistical significance. Statistical significance was considered at *p* < 0.05.

## Results

### ADK Inhibition Attenuated the Severity of SAP in Mice

**FIGURE 1:**
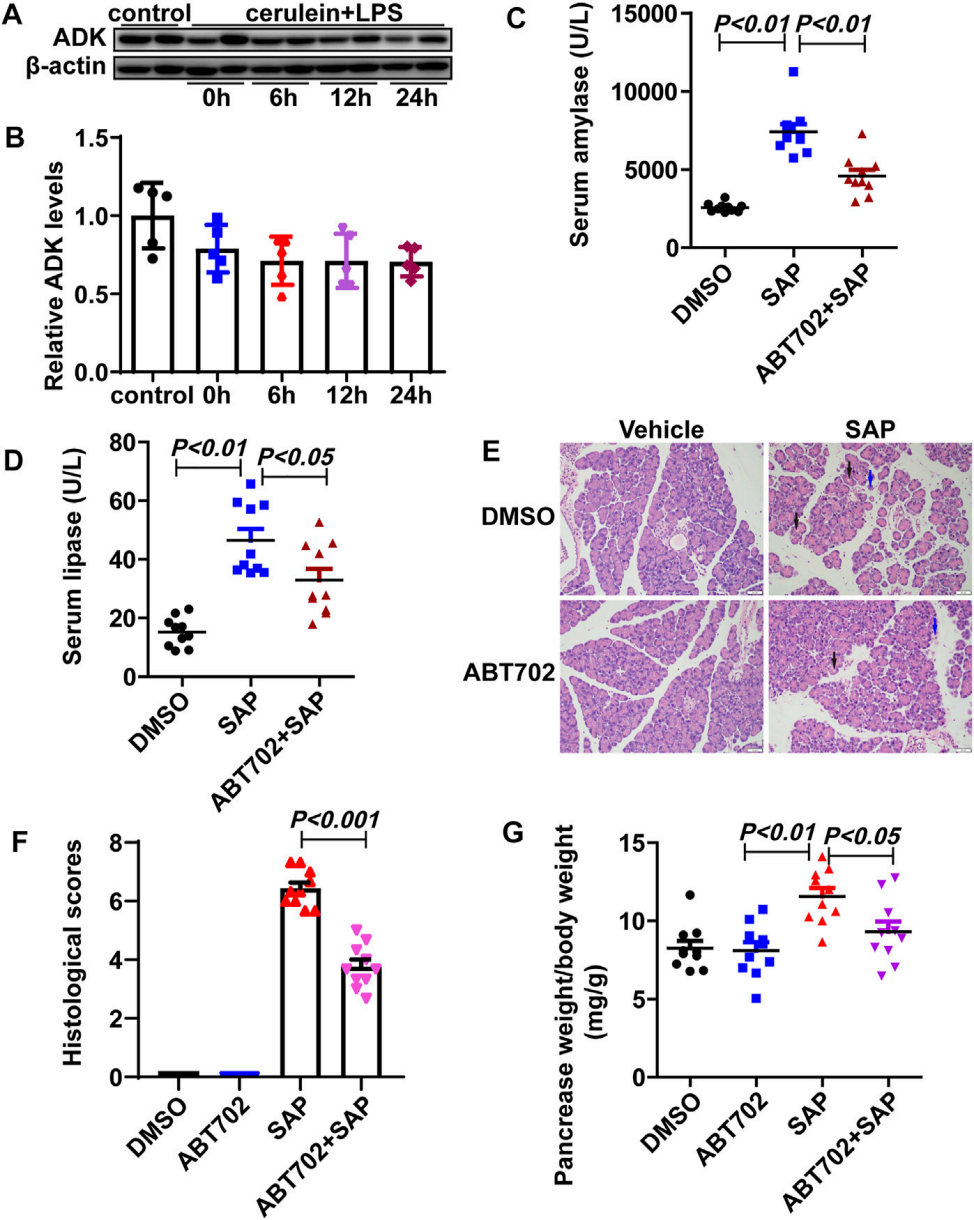
Effects of ADK inhibition on SAP in mice. **(A,B)**, The expression of ADK in pancreas from cerulein/LPS treated mice. (*n* = 5). **(C,D)**, Serum amylase and lipase were determined. (*n* = 10). **(E)**, HE staining of sections of pancreas. (*n* = 10). **(F)**, Histological scores were calculated referred to HE staining. Vacuoles: black arrows. Necrosis: blue arrows. (*n* = 10). **(G)**, The pancreas weight to body weight ratio was calculated. (*n* = 10).

The expression of ADK was not changed in the pancreas of SAP mice compared with that of control mice (Figures 1A,B). Nevertheless, ADK inhibition significantly prevented the increase in serum amylase (7,416.76 ± 1,457.76 vs. 4,581.89 ± 1,175.04 U/L) and lipase (46.51 ± 11.50 vs. 32.94 ± 11.46 U/L) in SAP mice (Figures 1C,D). Compared with the SAP group, pancreatic injury characterized by cytoplasmic vacuoles and tissue edema was alleviated in ABT702 - treated SAP mice (Figure 1E). Moreover, both the histological score (6.433 ± 0.60 vs. 3.77 ± 0.70) and the pancreas weight to body weight ratio indicated that ADK inhibition effectively limited the severity of cerulein + LPS—induced SAP in mice (Figures 1F,G).

ADK inhibition prevented the infiltration of inflammatory cells and reduced necroptotic response in the pancreas of SAP mice.

**FIGURE 2:**
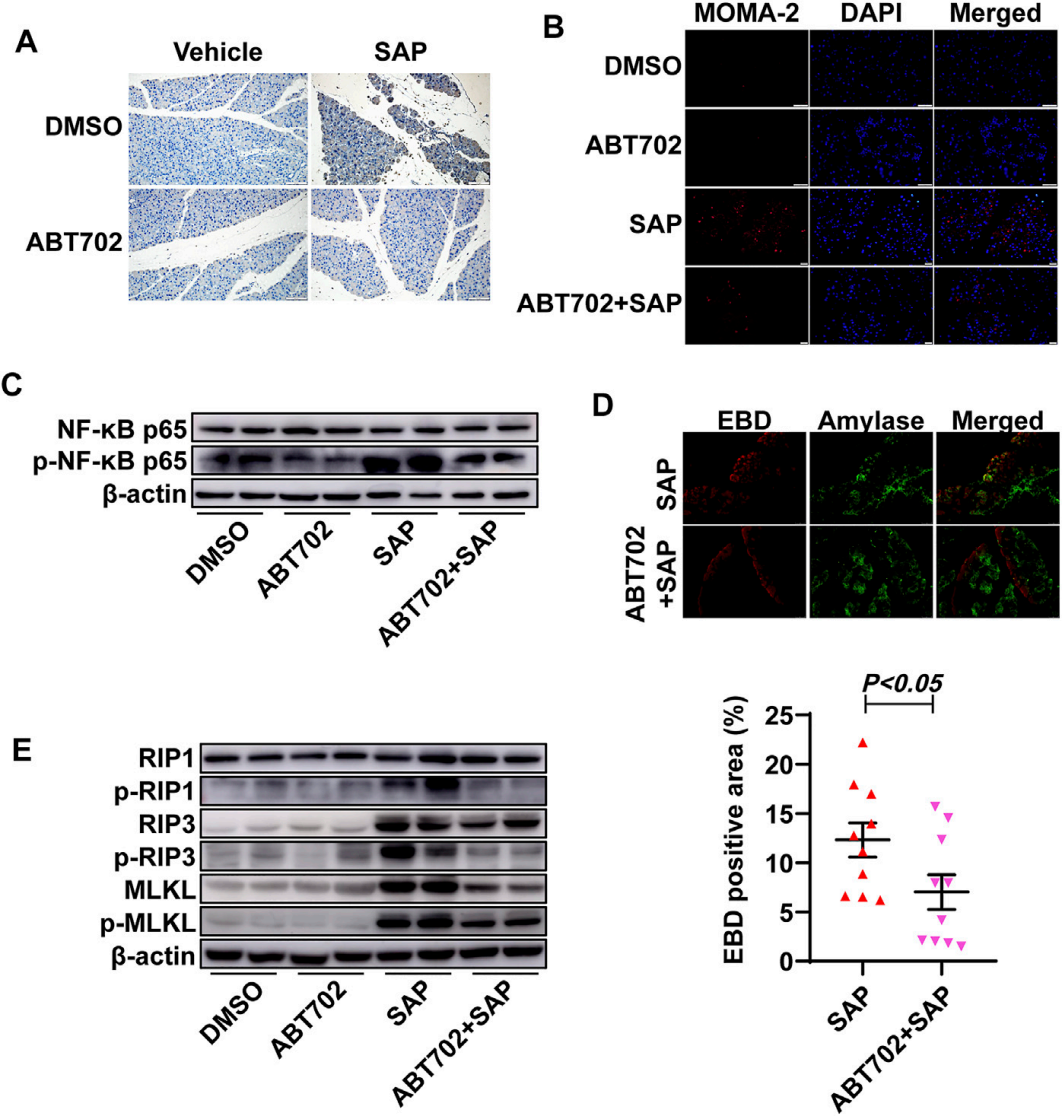
Effects of ADK inhibition on inflammation and pancreatic cell necrosis. **(A)**, Neutrophils were immunochemically staining using anti-CD11b antibody. (*n* = 10). Scale bar = 50 μm. **(B)**, Macrophages were visualized using immunofluorescence staining with anti-MOMA-2 antibody. (*n* = 10). Scale bar = 50 μm. **(C)**. NF-κB-P65 and the phosphorylation of NF-κB-P65 were immunoblotted. (*n* = 5). **(D)**, Pancreatic necrosis was visualized by double staining of EBD and anti-amylase. (*n* = 10). **(E)**, The critical molecules of the necroptotic pathway were immunoblotted. (*n* = 5).

Immunohistochemical staining showed that both neutrophils and macrophages in pancreatic tissue were significantly reduced in ABT702—treated SAP mice compared with those in SAP mice (Figures 2A,B, Supplemental Figures 1A,B). NF-κB regulates the transcription of >150 genes, many of which exhibit proinflammatory properties (Wei et al., 2013). Then, NF-κB activation as indicated by the phosphorylation of P65 in the pancreas was determined using immunoblotting. P65 was markedly phosphorylated in the pancreas samples from SAP mice and ABT702 effectively prevented this change (Figure 2C, Supplemental Figure 1C).

The area of the necrotic pancreas in the ADK inhibition group was noticeably reduced compared with that in the SAP mice (Figure 2D). As necroptosis is an important severity determinant in SAP, the necroptotic pathway was then detected (Louhimo et al., 2016). Cerulein + LPS treatment increased the expression of RIP3 and MLKL and promoted the phosphorylation of RIP1, RIP3, and MLKL. As expected, ADK inhibition reversed these changes in SAP mice (Figure 2E, Supplemental Figures 1D–I).

### ADK Inhibition Prevented Cerulein–Induced Inflammation and Cell Necroptosis in Acinar Cells

**FIGURE 3:**
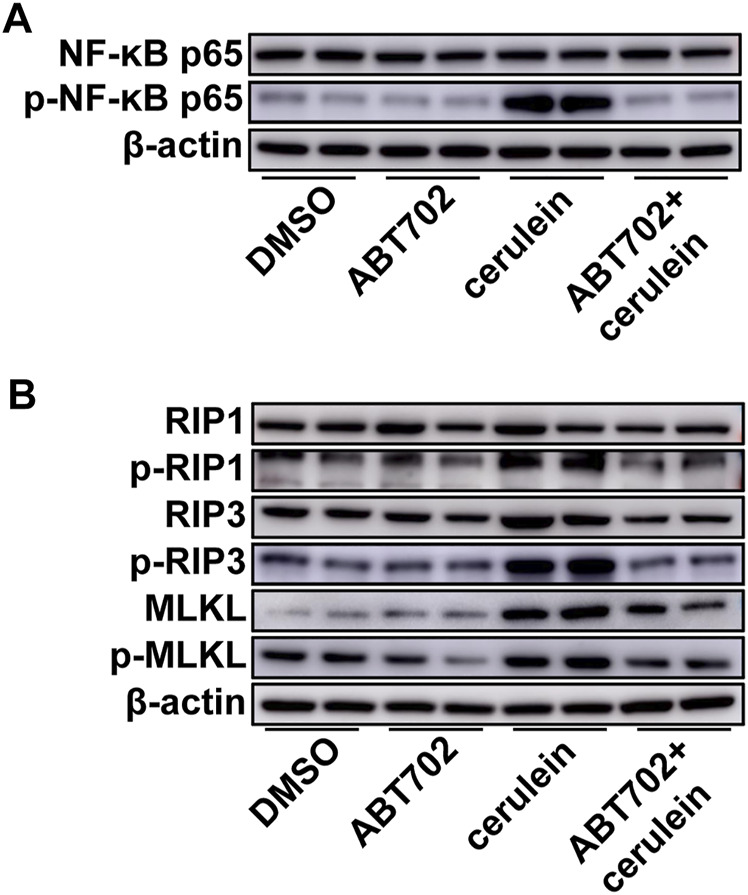
Effects of ADK inhibition on cerulein–induced inflammation and necroptosis in AR42J cells. AR42J cells were pretreated with ABT702 or DMSO and then exposed to cerulein for 24 h. **(A)**, NF-κB-P65 and the phosphorylation of NF-κB-P65 were immunoblotted. (*n* = 5). **(B)**, The critical molecules of the necroptotic pathway were immunoblotted. (*n* = 5).

Cerulein induced NF-κB activation in AR42J cells and ABT702 suppressed the effect of cerulein on NF-κB (Figure 3A, Supplemental Figure 2A). For cell necroptosis, we found that cerulein was able to increase the expression of RIP3 and MLKL and promote the phosphorylation of RIP1, RIP3, and MLKL *in vitro* (Figure 3B, Supplemental Figures 2B–G). Consistent with the *in vivo* results, ADK inhibition effectively prevented the activation of the necroptotic signaling pathway (Figure 3B, Supplemental Figures 2B–G). ADK inhibition showed similar effects on NF-κB activation and necropototic signaling pathways in cerulein—treated MPC-83 cells (Supplemental Figure 3).

### Endoplasmic Reticulum Stress Was Alleviated by ADK Inhibition

**FIGURE 4:**
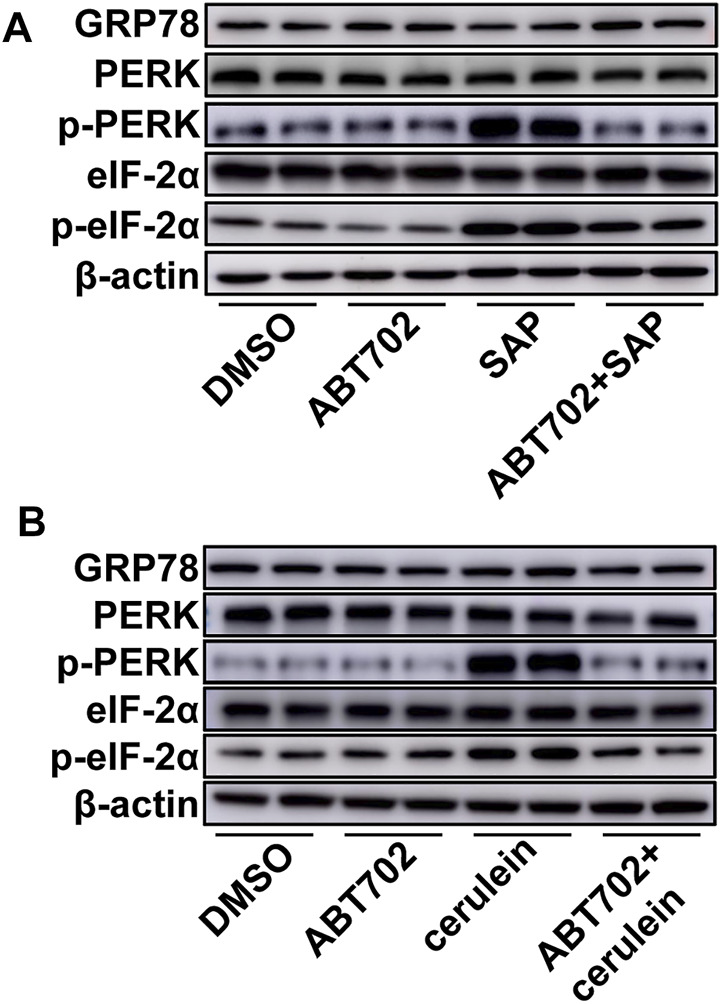
Effects of ADK inhibition on ER stress. Proteins were extracted from pancreatic tissues from SAP/control mice **(A)** or cerulein–stimulated AR42J cells **(B)**. GRP78, PERK, p-PERK, eIF-2*α* and p-eIF-2*α* were respectively immunoblotted. (*n* = 5).

Although GRP78, PERK, and eIF-2α were not changed, greater phosphorylation of PERK and eIF-2α was detected in the pancreas from SAP mice compared with control mice (Figure 4A, Supplemental Figure 4A–E). More importantly, we found that ADK inhibition decreased the phosphorylation of PERK and eIF-2*α* in the pancreases of SAP mice (Figure 4A, Supplemental Figures 4A–E). *In vitro* studies of AR42J cells also showed that ADK inhibition prevented cerulein–induced phosphorylation of PERK and eIF-2α (Figure 4B, Supplemental Figure 4F–J).

### ER Stress Contributed to Cerulein–Induced Cell Necroptosis *in vitro*

**FIGURE 5:**
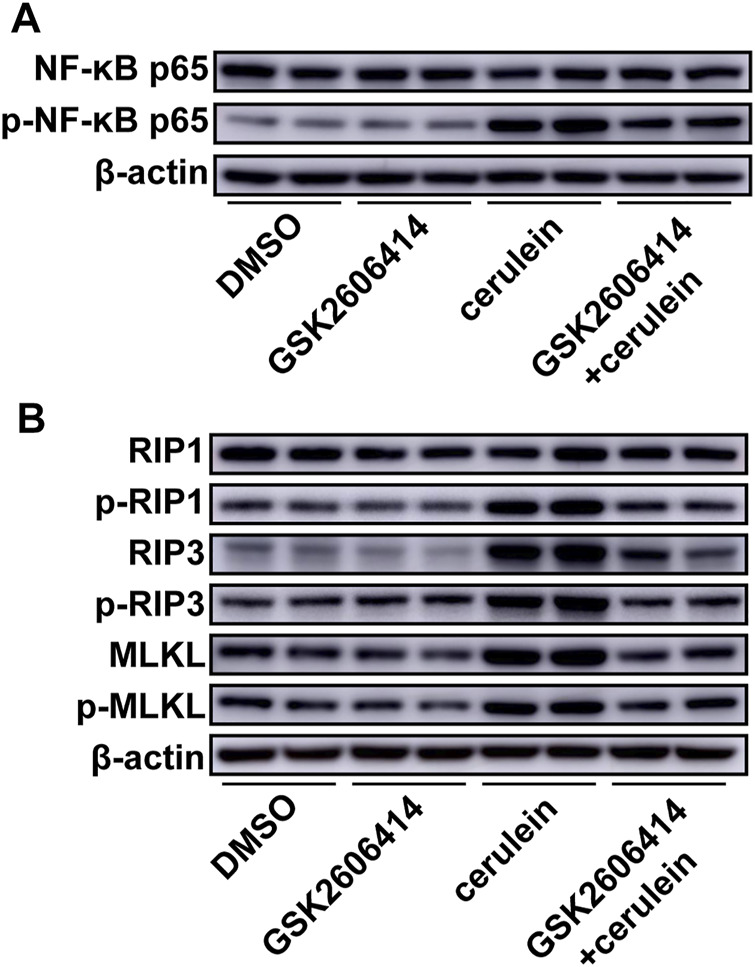
Effects of PERK inhibition on cerulein–induced inflammation and necroptosis in AR42J cells. AR42J cells were pretreated with GSK2606414 or DMSO and then exposed to cerulein for 24 h. **(A)**, NF-κB-P65 and the phosphorylation of NF-κB-P65 were immunoblotted. (*n* = 5). **(B)**, The critical molecules of the necroptotic pathway were immunoblotted. (*n* = 5).

PERK inhibition using GSK2606414 markedly reduced cerulein-induced phosphorylation of NF-κB (Figure 5A, Supplemental Figure 5A). PERK inhibition effectively prevented the increase in RIP3 and MLKL and reversed the upregulated phosphorylation of RIP1, RIP3, and MLKL under cerulein stimulation (Figure 5B, Supplemental Figure 5B–G).

Adenosine A_2A_ receptor mediates the effect of ADK inhibition on cerulein–induced inflammation and cell necroptosis.

**FIGURE 6:**
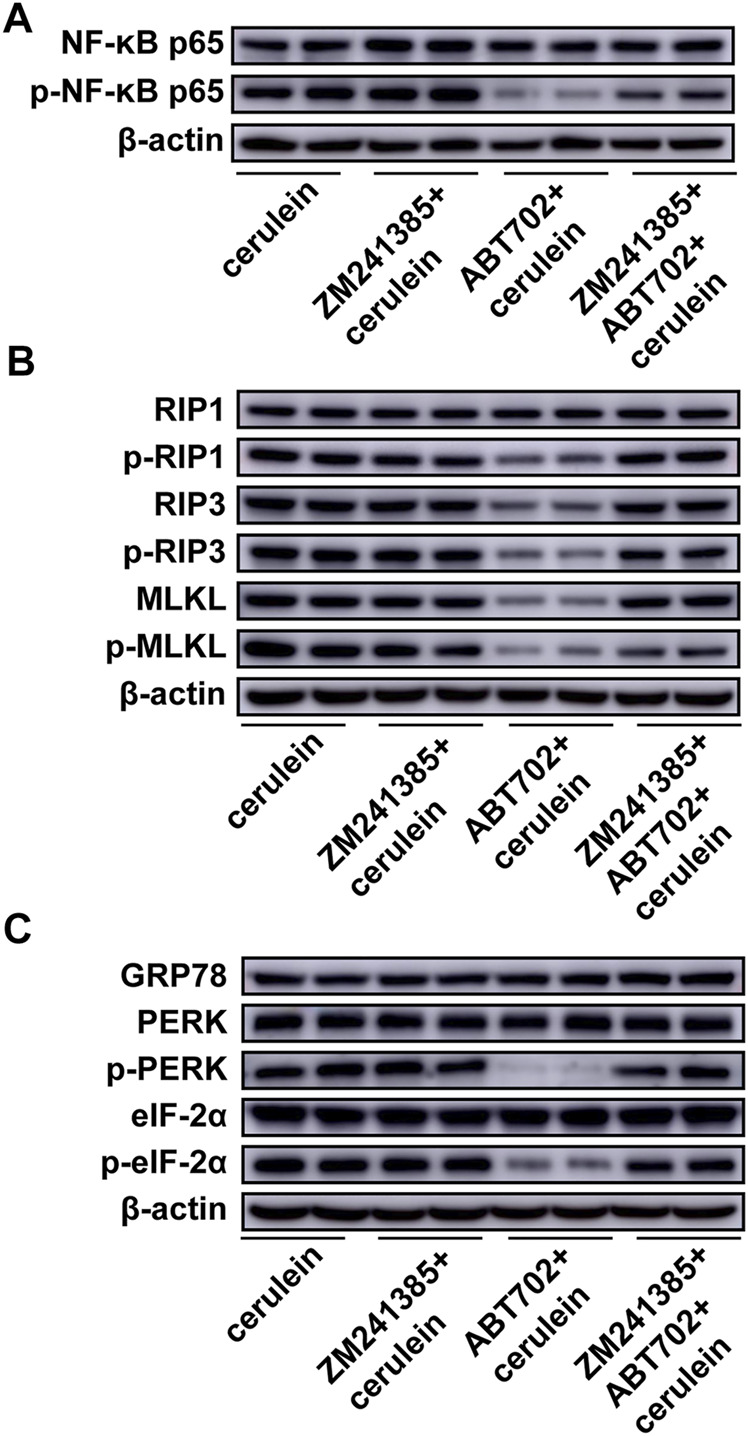
Effects of A_2A_ inhibition on cerulein—induced inflammation, necroptosis and ER stress in AR42J cells. AR42J cells was pretreated with ABT702 or ZM241385 + ABT702 and then was exposed to cerulein for 24 h. **(A)**, NF-κB-P65 and the phosphorylation of NF-κB-P65 were immunoblotted. (*n* = 5). **(B)**, The critical molecules of the necroptotic pathway were immunoblotted. (*n* = 5). **(C)**, GRP78, PERK, p-PERK, eIF-2*α* and p-eIF-2*α* were immunoblotted. (*n* = 5).

To determine whether the A_2A_ receptor contributes to the prevention of cerulein-induced inflammation and cell necroptosis by ADK inhibition in acinar cells, we pretreated AR42J cells with ZM241385 (an A_2A_ receptor antagonist) before ABT702 stimulation. We found that the adenosine A2A receptor antagonist reversed the effect of ADK inhibition on the inflammatory andnecroptotic pathways (Figures 6A,B, Supplemental Figures 6A,B). Moreover, the alleviating effect of ADK inhibition on ER stress was eliminated by the adenosine A2A receptor antagonist (Figure 6C, Supplemental Figure 6C).

## Discussion

**FIGURE 7:**
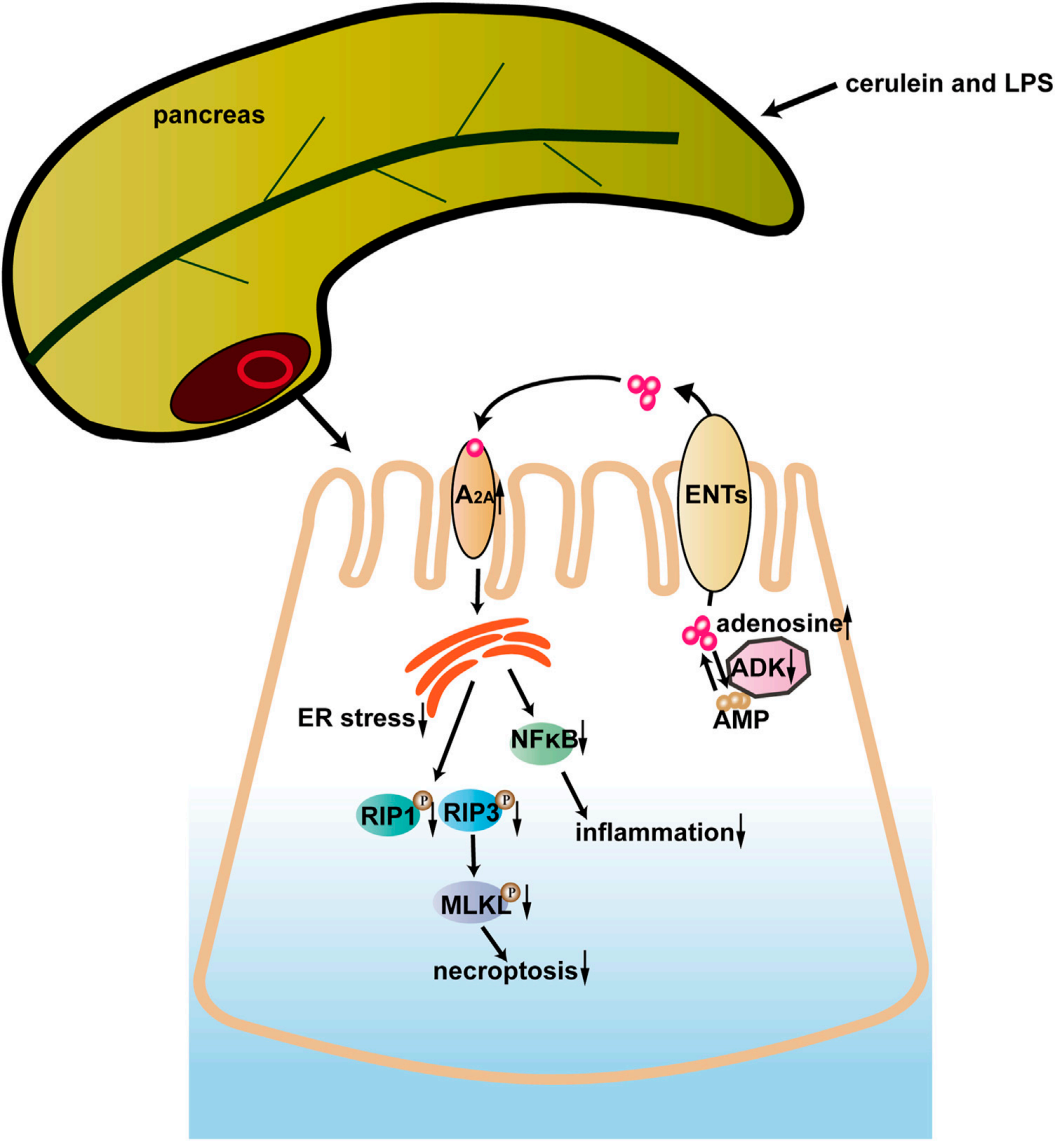
A model of the possible role of ADK in SAP.

In the current study, ADK inhibition alleviated cerulein + LPS—induced SAP by preventing the infiltration of neutrophils and macrophages, inhibiting the activation of the NF-κB pathway, and limiting necroptosis of acinar cells. ER stress played a critical role in the protective effect of ADK inhibition. Moreover, the adenosine A2A receptor might mediate the effect of ADK on ER stress, inflammation, and cell necroptosis. These data indicated that ADK might be a potential therapeutic target in SAP (Figure 7).

Adenosine is produced inside cells or on their surface, and it is always physiologically present at low levels both within and outside cells (Fredholm, 2007). The formation of adenosine rapidly increases in response to stress conditions and increased adenosine protects against cellular and tissue damage by acting on four G-protein coupled receptors: A_1_, A_2A_, A_2B,_ and A_3_ (Antonioli et al., 2013). The use of an adenosine uptake inhibitor showed that increased adenosine attenuated cerulein-induced acute pancreatitis (Noji et al., 2002). In the present study, we found that ADK inhibition showed protective effects against SAP. Adenosine should play a critical role in the effect of ADK inhibition on SAP because ADK is a determinant of adenosine levels. All four types of adenosine receptors are expressed in the mouse pancreas (Hayashi, 2019). Activation of the adenosine A_2A_ receptor decreased the inflammatory cell infiltration and acinar cell necrosis in acute pancreatitis and the mRNA level of A_2A_ was much higher than that of other receptors in the pancreas (Satoh et al., 2002; Celinski et al., 2006). Thus, it is reasonable to speculate that the adenosine A_2A_ receptor contributes to the effects of ADK inhibition on SAP, and our results supported this speculation.

Several forms of acinar cell death occur during SAP, namely, apoptosis, necrosis, pyroptosis, and ferroptosis (Kang et al., 2014; Fan et al., 2021). Apoptosis is mainly responsible for cell death in mild acute pancreatitis while necrosis accounts for most of the cell death in SAP (Bhatia, 2004a). Necrosis has traditionally been viewed as a form of passive cell death, although RIP-driven necrosis was recently identified and was commonly termed as necroptosis (Zhai et al., 2021). Necroptosis is activated by ligand binding to death receptors such as tumor necrosis factor-alpha receptor 1 (TNFR1) (Chan et al., 2015). The signal of death receptor ligation promotes autophosphorylation of RIP1 which is a vital upstream kinase of necroptosis that recruits and binds to RIP3 to form necrosomes (Li et al., 2012). Subsequently, RIP3 promotes MLKL phosphorylation and provokes the killing activity of MLKL. Finally, phosphorylated MLKL disrupts the plasma membrane to induce cell death (Meng et al., 2021). MLKL deficiency or RIP3 deletion plays a protective role in cerulein–induced acute pancreatitis (Wu et al., 2013; Louhimo et al., 2016). Moreover, inhibition of necroptosis with necrostatin reduces the severity of experimental mouse pancreatitis (Louhimo et al., 2016). Conversely, a recent study showed that RIP3 and MLKL—mediated necroptosis exerts protective effects in AP (Boonchan et al., 2021). In the present study, we found that ADK inhibition prevented the activation of the necroptotic pathway, thus supporting the detrimental role of acinar cell necroptosis in SAP.

AP is an inflammatory disorder of the exocrine pancreas and multiple inflammatory cells participate in tissue injury of the pancreas (Habtezion et al., 2019). The infiltration of neutrophils plays an important role in the early phase of AP and contributes to the activation of trypsinogen and progression to SAP (Habtezion, 2015; Habtezion et al., 2019). Macrophages are activated, differentiate into a proinflammatory phenotype and secrete inflammatory factors, thereby exacerbating the severity of pancreatic injury (Hu et al., 2020). Similar to inflammatory cells, acinar cells also contribute to the local inflammatory response in the pancreas and NF-κB activation induces the production of cytokines and adhesion molecules in acinar cells (Dios, 2010). Previous studies indicated that ADK regulates the inflammatory response under pathophysiological injuries (Ahmad et al., 2014; Xu et al., 2017). Similarly, we found that ADK inhibition reduced the infiltration of inflammatory cells in the pancreas and suppressed the activation of NF-κB in acinar cells.

One of the important mechanisms for AP is the unfolded protein response (UPR) which is induced by ER stress (Wu et al., 2016; Barrera et al., 2018). Activation of the UPR is initiated by three signaling proteins, namely, PERK, inositol requiring enzyme 1 (IRE1), and activating transcription factor 6 (ATF6) (Barrera et al., 2018). ER stress-induced UPR signaling promotes the production of proinflammatory molecules by activating several proinflammatory transcription factors such as NF-κB (Garg et al., 2012). Moreover, ER stress regulates both cell apoptosis and necroptosis through multiple mechanisms (Sano and Reed, 2013). In the present study, ER stress was proven to be activated in the experimental SAP pancreas and ADK inhibition prevented the activation of ER stress. PERK inhibition effectively reduced cerulein–induced activation of the necroptotic pathway, supporting the idea that ER stress mediates the role of ADK in SAP.

Taken together, the present study demonstrated that ADK inhibition protects against experimental SAP by limiting inflammation and reducing cell necroptosis via A_2A_/ER stress pathway.

## Data Availability

The original contributions presented in the study are included in the article/Supplementary Material, further inquiries can be directed to the corresponding authors.
