# Supplementary material for: Adenosine Kinase Inhibition Prevents Severe Acute Pancreatitis via Suppressing Inflammation and Acinar Cell Necroptosis
Source: Front Cell Dev Biol. 2022 Feb 23;10:827714. doi: 10.3389/fcell.2022.827714 (PMC8904929; doi:10.3389/fcell.2022.827714)
Supplement: Supplementary file 1 [file DataSheet1.docx]

**Supplementary data**

Supplemental Figures and Figure Legends:


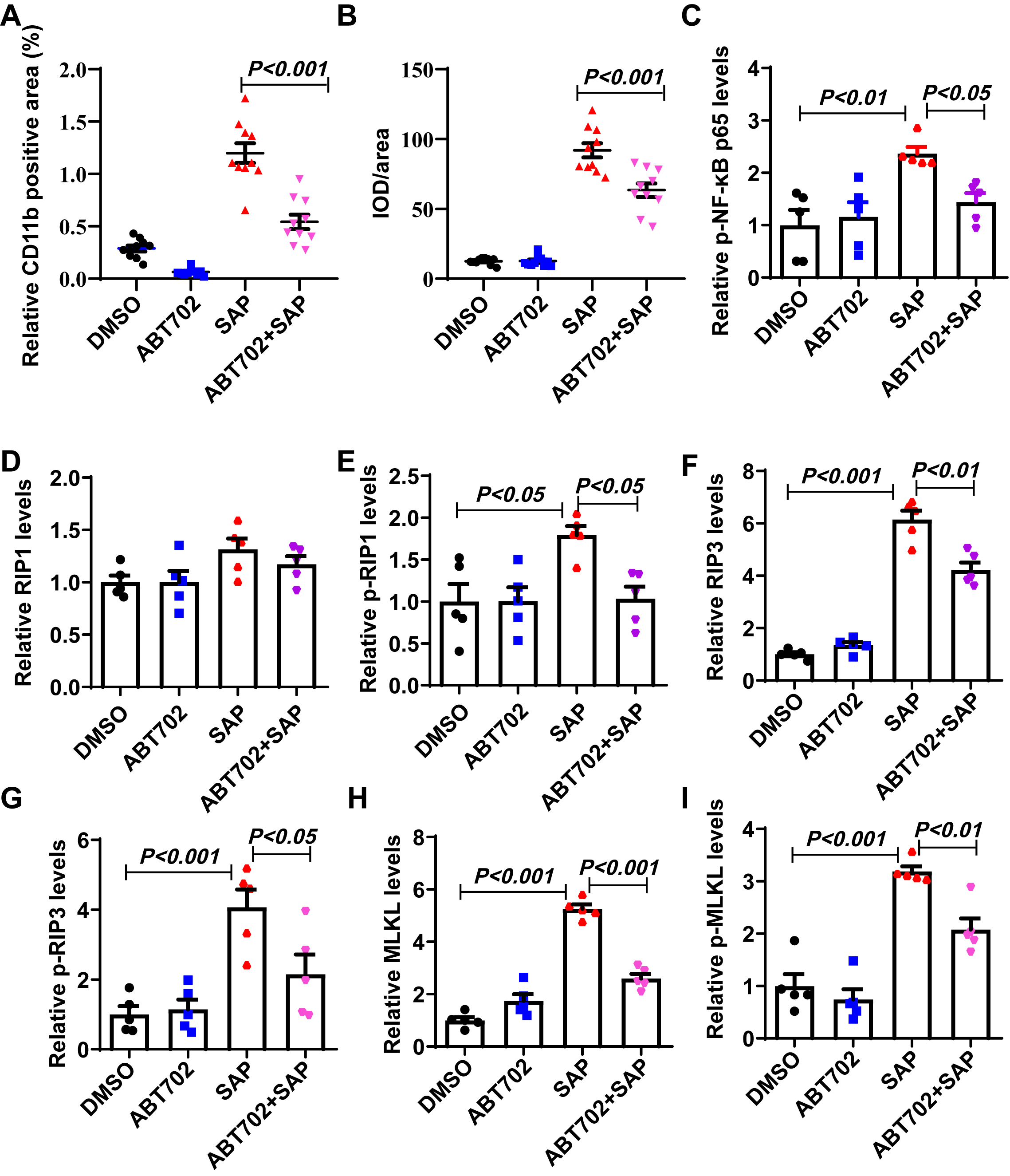


**Supplemental Figure 1. A,** Quantitative analysis of CD11b positive area in Figure 2A. **B,** Quantitative analysis of MOMA-2 in Figure 2B. **C,** Quantitative analysis of p-NF-κB in Figure 2C. **D-I,** Quantitative analysis of immunoblots in Figure 2E.


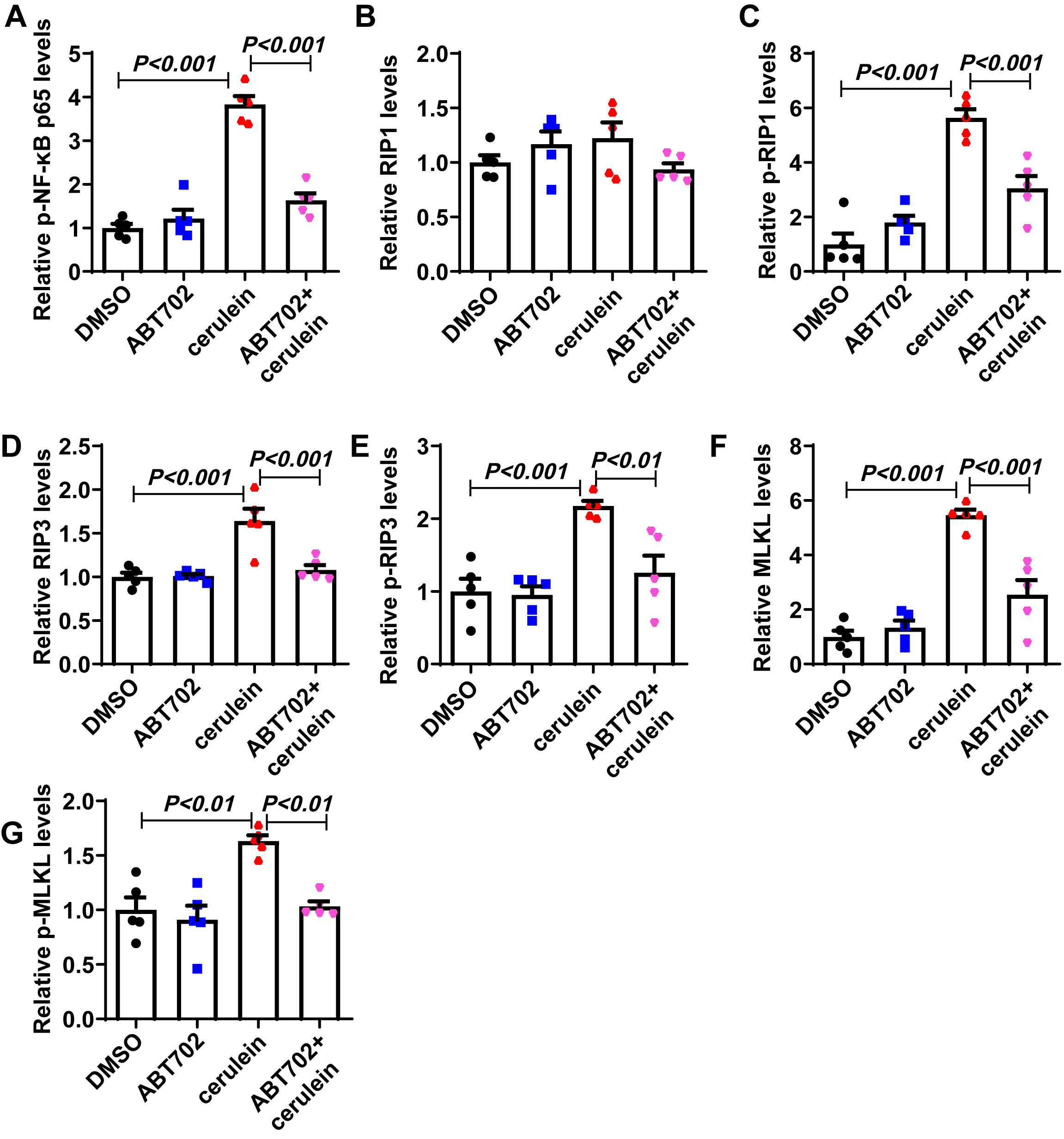


**Supplemental Figure 2.** **A,** Quantitative analysis of p-NF-κB in Figure 3A. **B-G,** Quantitative analysis of immunoblots in Figure 3B.


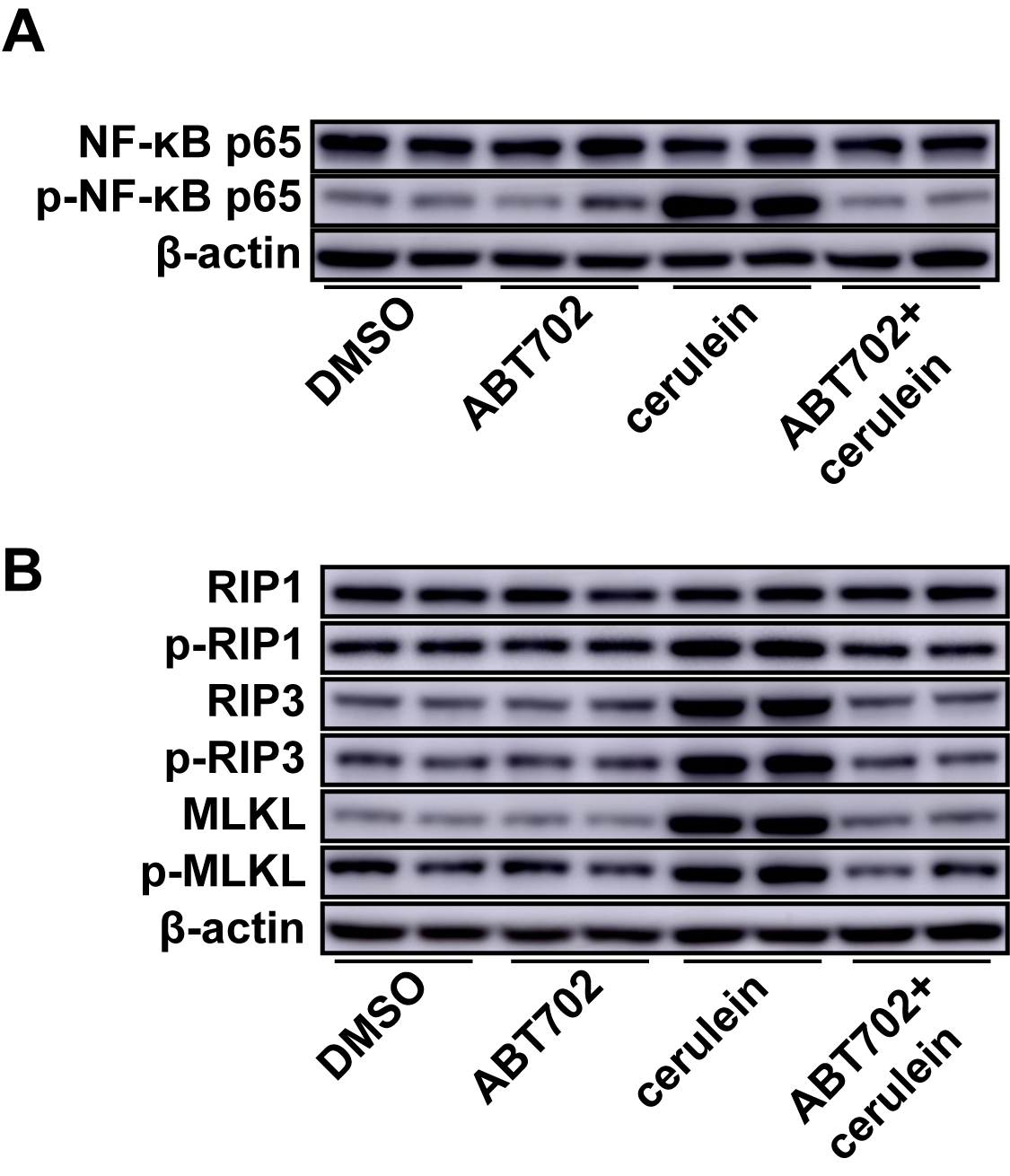


**Supplemental Figure 3**. Effects of ADK inhibition on cerulein–induced inflammation and necroptosis in MPC-83 cells. MPC-83 cells were pretreated with ABT702 or DMSO and then exposed to cerulein for 24 h. A, NF-κB-P65 and the phosphorylation of NF-κB-P65 were immunoblotted. (n=5). B, The critical molecules of the necroptotic pathway were immunoblotted. (n=5).


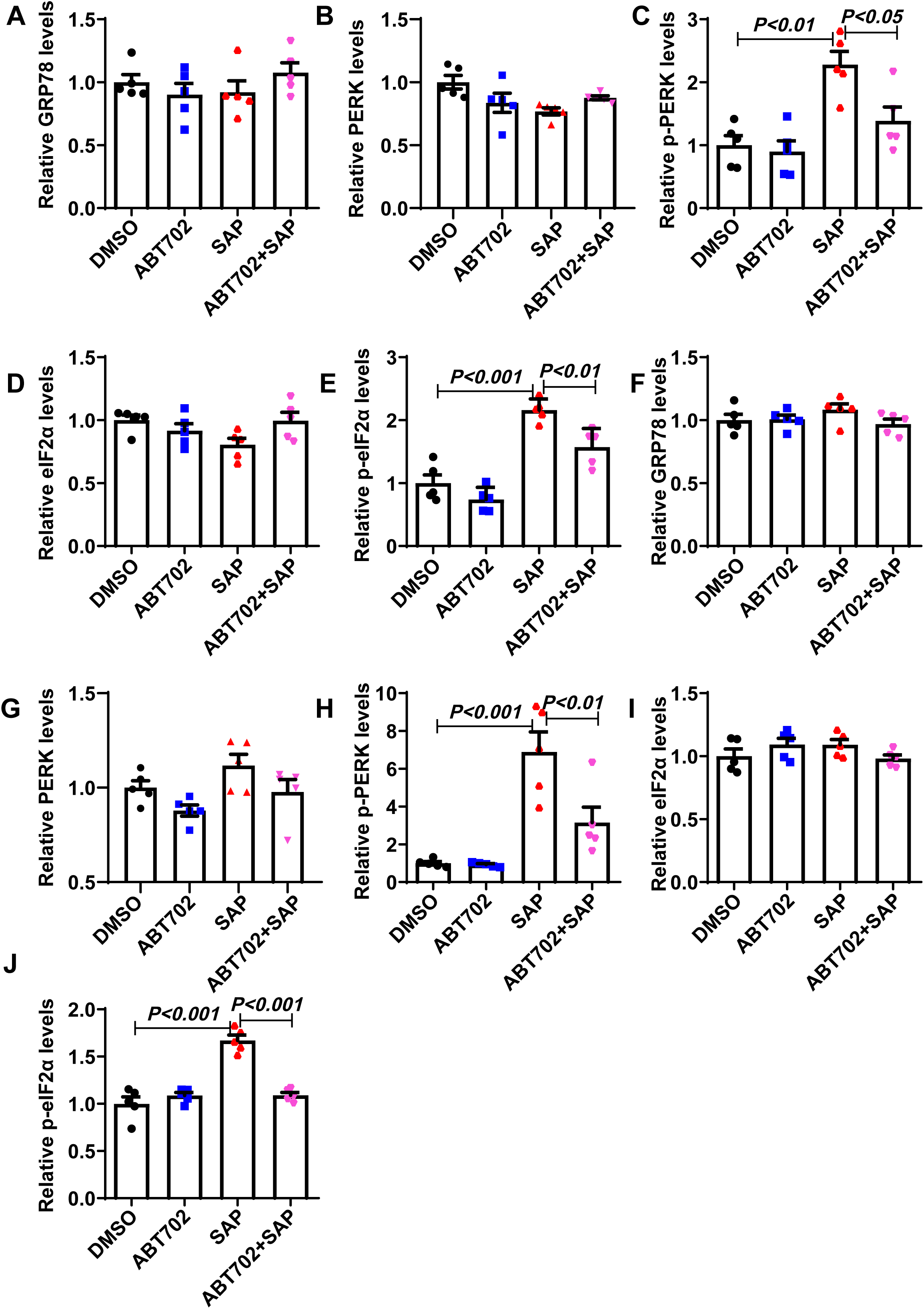


**Supplemental Figure 4.** **A-E,** Quantitative analysis of immunoblots in Figure 4A. **F-J,** Quantitative analysis of immunoblots in Figure 4B.


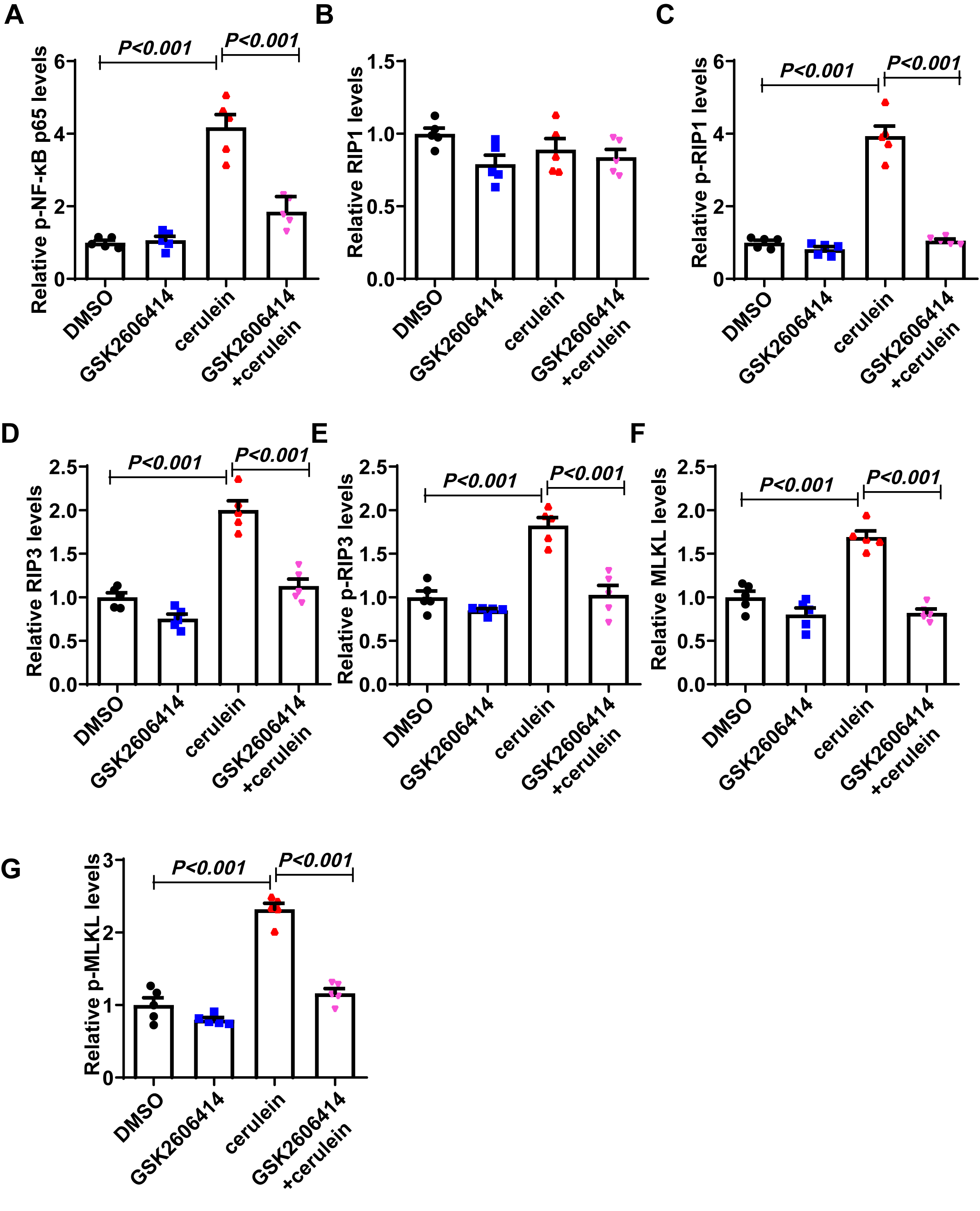


**Supplemental Figure 5.** **A,** Quantitative analysis of p-NF-κB in Figure 5A. **B-G,** Quantitative analysis of immunoblots in Figure 5B


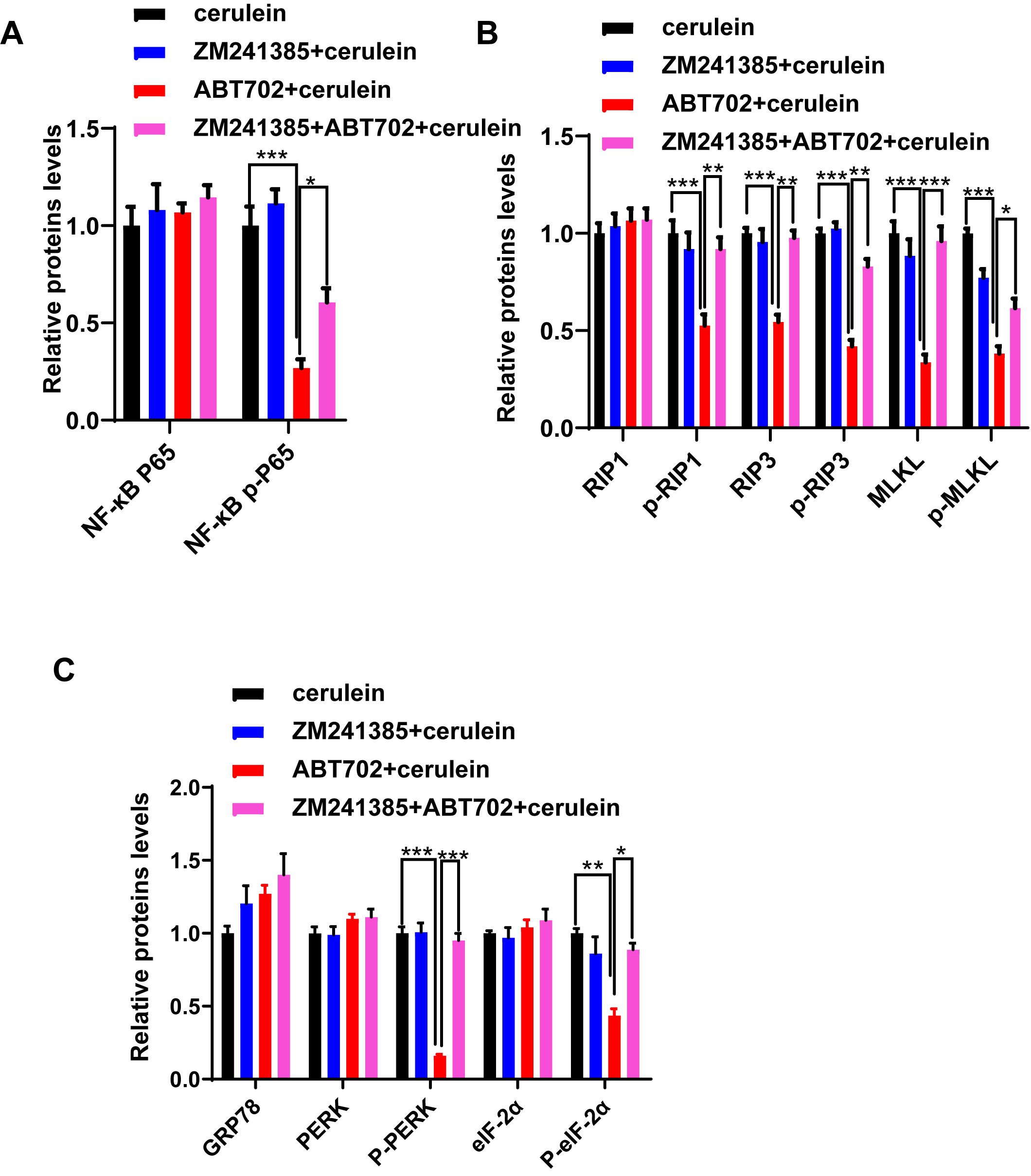


**Supplemental Figure 6.** **A,** Quantitative analysis of immunoblots in Figure 6A. **B,** Quantitative analysis of immunoblots in Figure 6B. **C,** Quantitative analysis of immunoblots in Figure 6C. **P*<0.05, ***P*<0.01, ****P*<0.001 vs control.
